# Integration of microbubbles with biomaterials in tissue engineering for pharmaceutical purposes

**DOI:** 10.1016/j.heliyon.2020.e04189

**Published:** 2020-06-17

**Authors:** Javad Esmaeili, Farnoush Sadat Rezaei, Farzaneh Mahmoudi Beram, Abolfazl Barati

**Affiliations:** a Department of Chemical Engineering, Faculty of Engineering, Arak University, Arak, Iran; b Histogenotech Co., R&D Department, Tehran, Iran; c Department of Chemical Engineering, Faculty of Engineering, Amir Kabir University, Tehran, Iran; d Department of Chemistry, Faculty of Chemistry, Isfahan University, Isfahan, Iran

**Keywords:** Biomedical engineering, Bioengineering, Chemical engineering, Biochemical engineering, Drug delivery, Nanoparticles, Microbubble, Biomaterial, Tissue engineering, Scaffold, Oxygen

## Abstract

Tissue engineering with the aid of biomaterials is a novel and promising knowledge aiming at improving human life expectancy. Besides, microbubbles are increasingly employed in biomedical applications due to their capability as a reservoir of therapeutic agents and oxygen molecules. In the present study, Microbubbles as the backbone of the research are produced as one of the potent devices in tissue engineering approaches, including drug delivery, wound healing, 3D printing, and scaffolding. It was shown that microbubbles are capable of promoting oxygen penetration and boosting the wound healing process by supplying adequate oxygen. Microbubbles also demonstrated their strength and potency in advancing drug delivery systems by reinforcing mass transfer phenomena. Furthermore, microbubbles developed the mechanical and biological characteristics of engineered scaffolds by manipulating the pores. Increasing cell survival, the biological activity of cells, angiogenesis, cell migration, and also nutrient diffusion into the inner layers of the scaffold were other achievements by microbubbles. In conclusion, the interest of biomedical communities in simultaneous usage of microbubbles and biomaterials under tissue engineering approaches experiences remarkable growth in Pharmaceutical studies.

## 1 Introduction

One of the exciting approaches amongst medical communities and scientists is employing microbubbles (MBs) (Box 1) in their researches and studies due to its unique potentials such as promoting drug/gene/oxygen delivery, blood substitute, and ultrasound agents. On the other hand, Tissue Engineering (TE) (Box 2), as a multidisciplinary science, has experienced growing interest among scientists. TE provides various options in different fields of science, including drug delivery, drug testing, wound healing, 3D media and models, cell culturing, drug approval, tissue regeneration, regenerative medicine, and so on. Incorporating MBs into TE has made progress in pharmaceutical and numerous treatments like cancer therapy, tissue regeneration, implants, and wound healing. Like other methods in medicine, TE still faces challenges and weaknesses. It has been proved that MBs can amplify and amend some of these issues due to their unique characteristics.

Box 1 **Microbubbles.**

**Figure 1:**
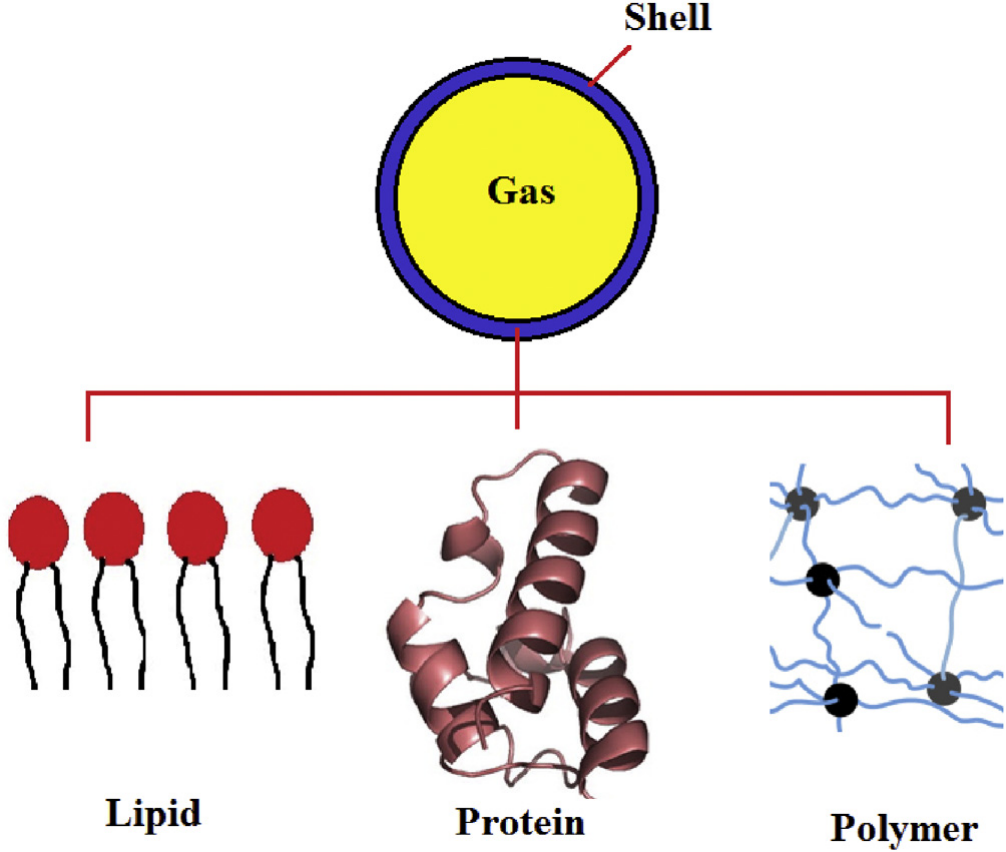
Schematic of microbubbles and the shell types that are used.

Microbubbles (Colloidal bubbles) are tiny gas bubbles in microscale (<50 μm) in diameter in liquids, containing oxygen, air, nitrogen, perfluoropropane, perfluorobutane, perflurohexane, sulfur hexafluoride, and other gases. Also, they stay relatively stable and steady in water for an extended period but also they rise very stilly and slowly. Microbubbles are made of a gaseous core and a shell that can be made from various kinds of materials, including Protein, Surfactant, Lipid, Polymer, and Polyelectrolyte Multilayer, Figure 1 (nicely reviewed by S Sirsi [1]).

Alt-text: Box 1

Box 2 **Tissue Engineering.**

There had always been a desire to have a long and healthy life. However, losing an organ, damaged tissue, cancer, and other diseases decrease the possibility for this hope. TE opens new doors to people by enhancing the chance of regeneration of the damaged or lost organs and also creating novel therapies or advancing the current treatments. TE is combining cells, materials, and engineering. It consists of three factors: cells, signals, and scaffolds. With the aid of TE, it is possible to repair a damaged portion of a tissue or replace the whole organ, Figure 3 [17].

Alt-text: Box 2

Despite these, according to our appraisal, fewer studies can be found in literature considering the employment of MBs in TE While MBs have been incorporated in other studies copiously.

### 1.1 Potential clinical applications of microbubbles

**Figure 2:**
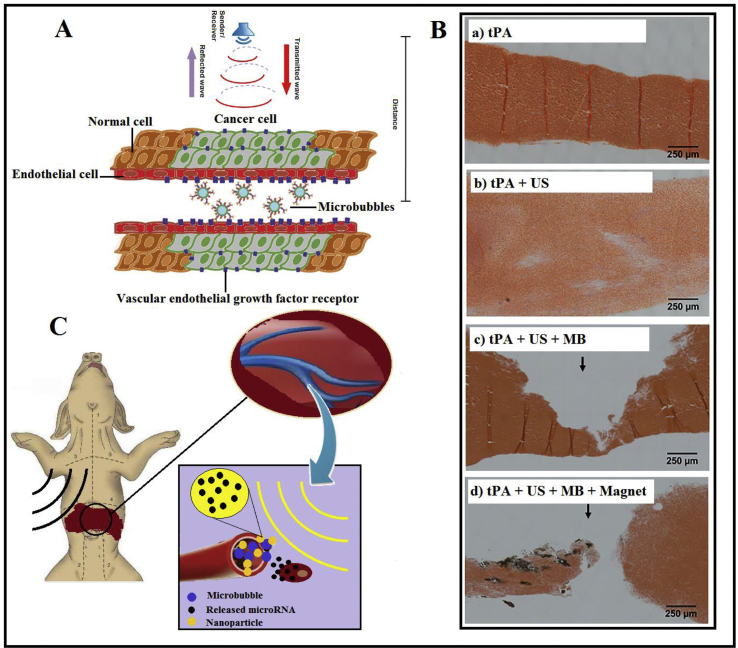
A) Ultrasound molecular imaging with MBs targeting cancer cells. B) The effect of employing MBs for clot removal in the absence and presence of the magnetic field (US = ultrasound; tPA = tissue plasminogen activator) [6]. C) Illustration of ultrasound-mediated drug delivery platform with the aid of MBs.

MBs revealed numerous noticeable effects during combination with conventional therapies. For instance, MBs were employed for pharmaceutical removal in water with the aid of ozonated MBs [2]. Besides, MBs are known as significant contrast agents due to their high compressibility for imaging [3]. **Ultrasound Molecular Imaging** (see the glossary) provides data about markers that are beneficial for cancer diagnostics. MBs functionalized with targeting ligands help ultrasound imaging to work effectively for cancer **angiogenesis** imaging (Figure 2A) [4].

**Ischemic stroke** still is known as one of the main reasons for inability and fatality worldwide. MBs showed their ability to fight against **Thrombosis** in combination with ultrasound [5]. Figure 2B shows the effect of MBs on clot removal efficacy. As can be seen, decreasing the clot diameter for the experiments revealed the positive impact of MBs targeting upon **clot lysis** rates in the absence of a magnetic field [6].

Drug/gene carriers are capable of being co-injected or coupled directly to MBs to achieve higher therapeutic efficacy [7]. Also, MBs proved to be the right candidate for guided delivery of drugs [8], nucleic acid [9], therapeutic gas (hydrogen sulfide, nitric oxide, oxygen, and carbon monoxide) [10] and short interfering RNA (siRNA) [11]. In a similar study, the power of MBs has demonstrated the delivery of miRNA-loaded nanoparticles (safely and noninvasively) to deep tissues in big animal models like a pig [12], as can be seen in Figure 2C, MBs under the ultrasound beam, augment the vascular diffusion and enhance nanoparticle extraversion, which leads to better drug delivery.

Having a high surface to volume ratio makes MBs enable to promote the transport phenomena. For instance, in 2018, it was demonstrated that MBs could help oxygen transport to improve breast cancer therapy by increasing the concentration of oxygen within the breast tumor, which resulted in slowing breast tumors growth rate in the mice. In this work, it was approved that MBs make tumors more sensitive to radiation (three-times) [13].

Tumor physiology alteration, making cells more permeable to oxygen and **chemotherapy** as well, are the benefits of MBs in cancer therapy [13]. Likewise, SM Fix et al. [14] showed the success of MBs in **ameliorating** the radiotherapy tumor control in a rat **fibrosarcoma** model. MBs size and also the pulse amplitude affect the efficiency of the MBs [15]. The size of the MBs can be minimized through oscillation frequency control [16].

**Figure 3:**
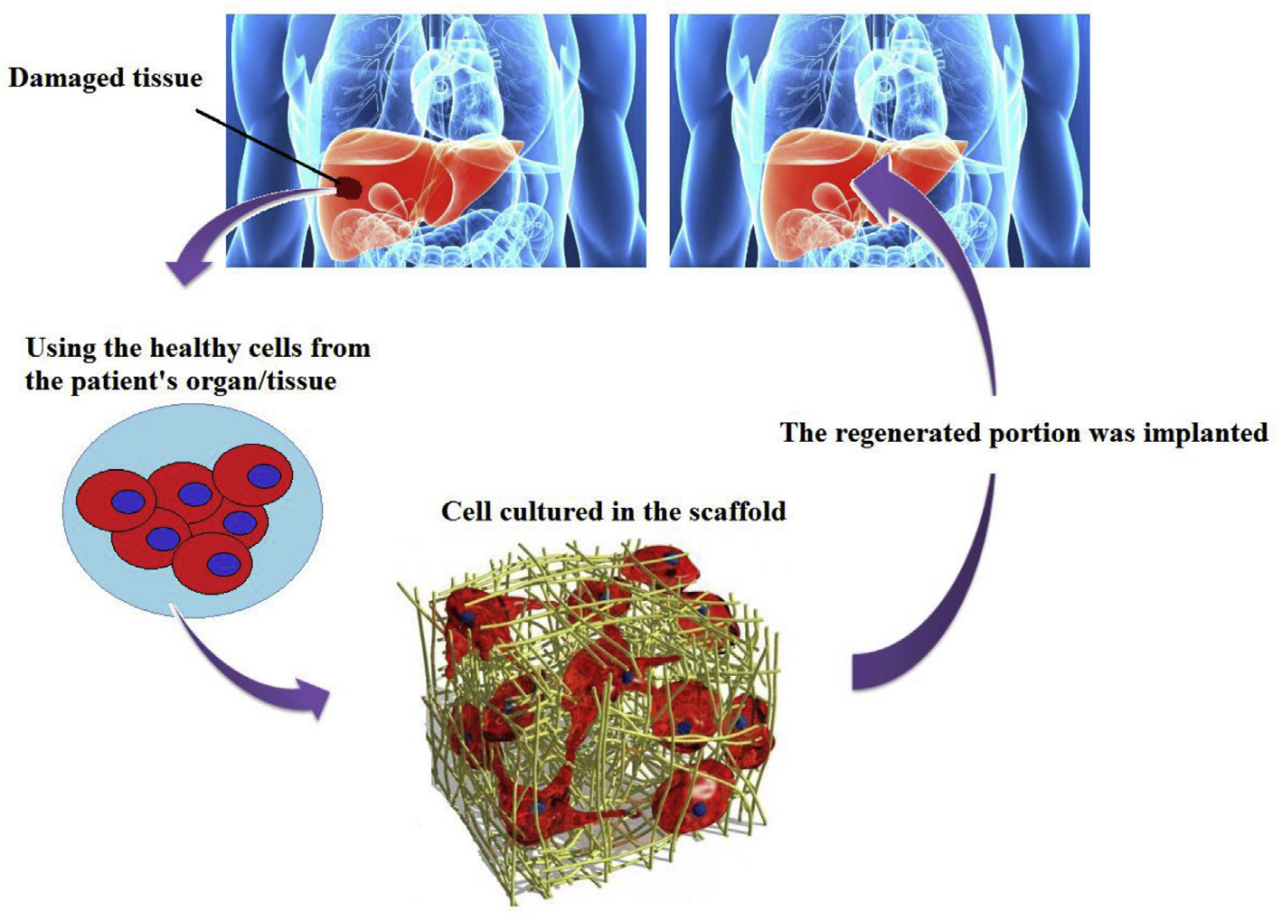
Schematic of Tissue Engineering process.

Local agitation caused by the MBs stream can be useful in the case of the **blood-brain barrier.** In this case, MBs are employed for opening the blood-brain barrier for targeted macromolecular agent delivery to the brain [18, 19]. Besides, in the case of gene delivery and **transfection,** crosslinked positive MBs based on **Bovine Serum Albumin** proved to be a good representative (nicely studied by Du) [20].

However, further studies about MBs employment in clinical applications can be found in the literature. Similarly, scientists in TE and biomedical engineering have been interested in MBs in combination with biomaterials. Despite fewer publications regarding this issue, in this study, we have tried to present a brief report about this issue by considering the various aspects including Wound healing, Drug delivery, Microfluidic devices (MFDs), Scaffolds and Fabrication. The main aim is to determine if MBs can affect the interaction of biomaterial with biological systems for a medical purpose.

## 2 Oxygen for wound healing

Wound healing involves the stages of blood clotting, angiogenesis, and skin repair. This process occurs well when growth factors (GFs) and cytokines are supplied. Therapeutic agents, including oxygen, GFs, cytokines, antibacterial agents, proteins, and bioactive agents, accelerate the wound healing process [21]. Oxygen is one of the main factors in wound healing, has always attracted researchers' attention (Table 1). Sufficient oxygen is of particular importance because of the demand for more biological energy to support compensative processes, including cell proliferation, angiogenesis, collagen synthesis, and bacterial defense. In light of the above, researchers in wound healing have been looking for adequate oxygen compensation at the wound site to accelerate wound healing. In 2019, in a review paper written by Chouhan et al. [22], a complete report regarding the novel and innovative approaches for wound healing and skin regeneration was presented. However, unfortunately, there was no mention of microbubbles and how they affect wound healing.

**Table 1:** Different methods to deliver oxygen.

Type of oxygen delivery system	Case of study	Ref.
Transdermal continuous oxygen therapy	Chronic Leg Ulcers	[25]
H_2_O_2_-induced ROS microenvironment	General wound healing process	[26]
Vacuum	Negative pressure wound therapy	[27]
Exosome laden oxygen	Antibacterial cryogel wound dressing OxOBand for diabetic and infectious wound healing	[28]
Photosynthetic sutures	General wound healing	[29]
Topical oxygen treatment	General wound healing	[30]
Oxygen-generating alginate hydrogel	Facilitating wound healing	[31]
Oxygen delivery dressings	Facilitating wound healing	[32]
Oxygen-generating film	Local wound oxygenation	[33]
Metal–Organic Frameworks (MOFs)	Bacterial infected wounds and environmental disinfection	[34]
MOFs	Wound Healing	[35]

### 2.1 Microbubbles

One of the essential characteristics of MBs is that they can inherently be made of oxygen, which not only it is not toxic and harmful to the human body, but also is one of the main factors in the survival of cells. Carbon nanotubes (CNTs) not only attracted the attention of experts in wound healing but also have been increasingly employed in biotechnology and biology [23], while the toxicity of CNT is still challenging. In a study by Zhang et al. [24], it was approved that CNT treated by acid ended up in adverse effects on cell physiological functions including cell adhesion, migration, spreading, and wound healing ability, while in another research incorporating CNT into wound dressing results in controlling delivery of antibiotics and antiseptics [36]. CNT induces reactive oxygen species, but they cannot be used as an oxygen carrier, because there is a likelihood of the carbon oxidation. On the other hand, there have been efforts to develop novel oxygen carriers by employing CNT. Coupling hemoglobin to functionalized multi-wall CNT (MWCNT) was introduced as a new oxygen carrier. The results depicted that heparin-conjugated MWCNT functionalized with hemoglobin by providing enough oxygen to the cells showed the highest cell viability [37].

To sum up, CNT must be functionalized with suitable groups for drug delivery (oxygen) purposes, while MBs seem to need fewer steps to carry oxygen or any drugs. They are easy to work when compared with CNT. Besides, they do not have cytotoxicity, mainly when employed along with biomaterials.

In the case of liposomes, an essential difference between MBs and liposomes is the gas loading capacity. MBs are made up of a gaseous core and have a higher loading capacity than liposomes.

Liposomes can only transfer around 10% gas by volume S.L. Huang, D.D. McPherson, R.C. Macdonald, A method to co-encapsulate gas and drugs in liposomes for ultrasound-controlled drug delivery, Ultrasound Med.Biol. 34 (2008) 1272–1280. To deliver NO and Xe as potent bioactive gasses, this amount of low loading capacity is sufficient. In contrast, for delivering oxygen, which is typically required in high concentrations for a therapeutic effect, liposomes are not more appropriate. Furthermore, there is a difference between MBs and ELIPs because of their size and their potential to extravasate; thereby, liposomes and MBs can be conjugated in order to reach higher efficiency in pharmaceutical purposes [38].

MBs are known as suitable carriers for maintaining and releasing oxygen when necessitates. Delivering drugs with adequate oxygen not only helps tissue to self-regenerate but also strengthens the cells to perform more appropriately. In some cases, MBs are employed directly, while sometimes a carrier must be used, which MBs carrier is another challenge.

**Figure 4:**
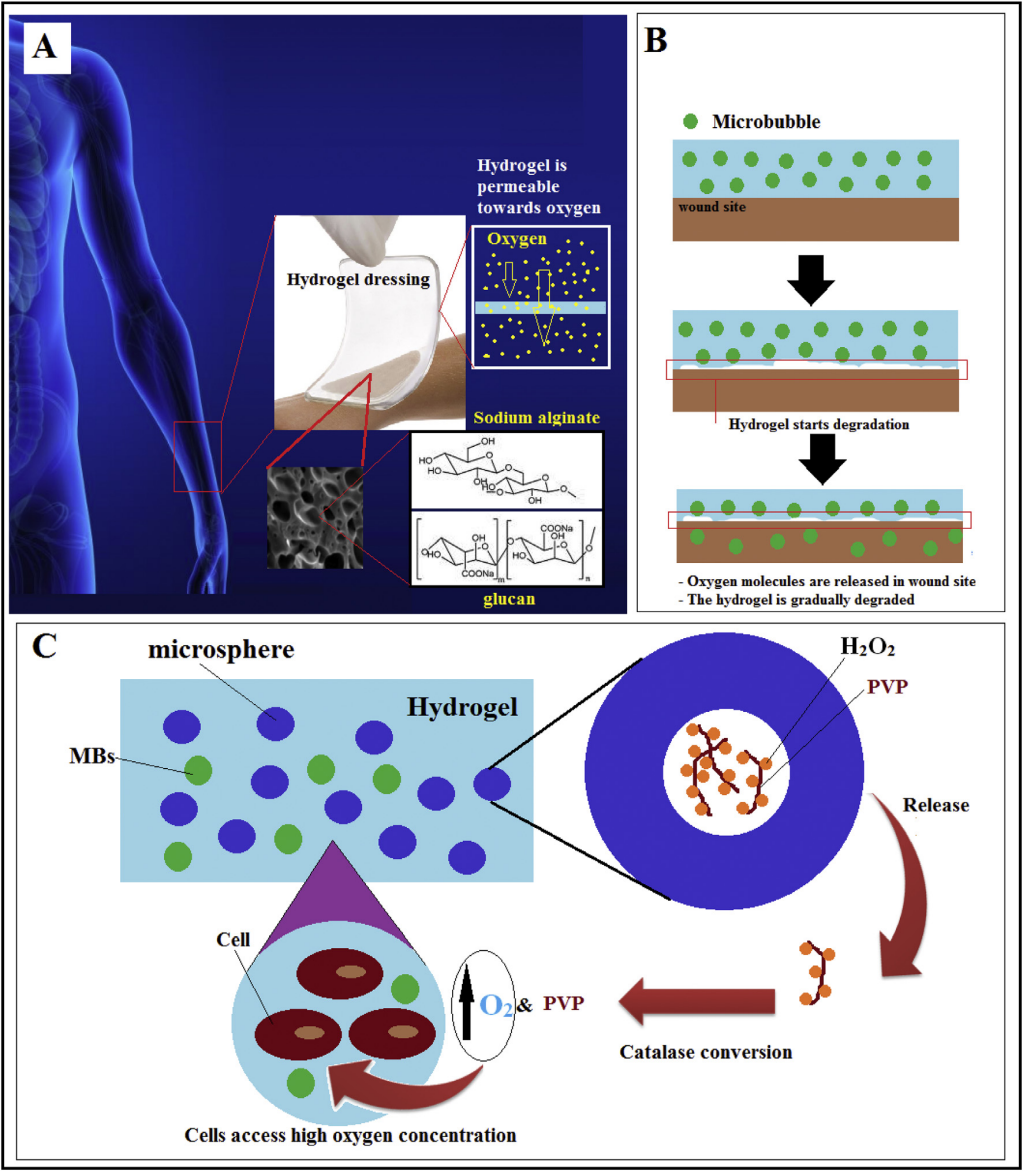
A) hydrogel-based wound healing. Hydrogels can be engineered to be receptive to O2. Hydrogels can be made by various biomaterials like sodium alginate and glucan. B) Hydrogels can be degraded during the healing process and release the embedded MBs. C) reaching a high concentration of oxygen by hyperbaric oxygen-generating hydrogel and MBs, PVP: Poly (vinyl pyrrolidone).

While TE deals with different wound treatment, embedding MBs with oxygen core within a porous hydrogel patch is known as a novel therapy. MBs are scattered in the hydrogel, and then the hydrogel is polymerized, which modifies the physical properties (mechanical properties and diffusivity) of hydrogels (Figure 4A), [39]. Hydrogels are also introduced to keep the moisture content at the proper level for wound healing. Moreover, hydrogels can be programmed [40] to be degradable or biocompatible in a specific manner during wound healing and then release MBs to the wound site (Figure 4B).

Making a cover capable of delivering high oxygen levels while maintains the necessitated moisture has attracted the attention of scientists. As a suitable depot and coating layer, hydrogels have made it possible for new bandages to not only become permeable but also provide oxygen to wounds (Figure 4A).

Furthermore, MBs are a potent device to increase nutrient transport and accelerate signaling. MBs can be integrated with biomaterials in two ways: i) polymeric-shelled MBs [41], and ii) MBs be locked inside a polymeric structure like hydrogel [42].

In light of the importance of oxygen in wound healing, scientists have been looking for different ways to supply oxygen. **Hyperbaric oxygen therapy**, on the other hand, is a new way of utilizing high-pressure oxygen for several specific wounds, such as radiation injuries, infections, burns, scratches and cuts, and diabetes mellitus, which cannot be healed with the conventional treatment modalities [43, 44]. This method uses oxygen at a pressure higher than sea level pressure. Various mechanisms have been employed in order to increase the concentration of oxygen at the wound site or even tumor location. Generally, the typical delivery/release mechanism of drugs, oxygen, and growth factors trapped in the MB, start with shell degradation. The outer layer of the shell starts degrading during the treatment, which helps the MB content to diffuse into the environment.

In some cases, MBs are entrapped inside a bio-polymeric bulk in which their contents are released while the bulk degrades. In contrast, in other cases, MBs can be released under a burst-release (second state of integration of MBs with biomaterials). This mechanism can be carried out by designing a carrier (e.g. Hydrogel) capable of bulk degradation.

For instance, in one study, Kang et al. [31] could engineer a hydrogel-based reservoir that can generate oxygen. They synthesized a hydrogel made of **Thiolated gelatin** that was a hyperbaric oxygen-generating hydrogel which rapidly produces O2 up to the hyperoxia levels. According to Figure 4C, in this study, core-shell microspheres were locked within the hydrogel. The core and shell were made of H_2_O_2_/Poly (vinyl pyrrolidone) complex and poly (lactide-co-glycolide). H_2_O_2_ molecules were decomposed into oxygen—this strategy allowed sustained oxygen delivery for at least 14 days. MBs combination with this technology augments the potential of this strategy and prolongs oxygen delivery.

Camci-Unal et al. [45] approved oxygen-releasing systems like hydrogels will stunningly augment cell viability and tissue reconstruction in the upcoming studies. They also reported that oxygen could be released in the form of MBs or by specific reactions (Figure 4C) [46].

The oxygen release system designed by Li et al. [47] showed its significant potential to improve the efficiency of cardiac stem cell therapy. Based on their results, the engineered oxygen-releasing system could noticeably reinforce and improve cell survival, and no death was observed during cell culture (after seven days). Also, cells were able to generate and grew during the period.

In another study done by Alemdar et al. [48], a calcium peroxide-based oxygen-generating hydrogel was created. They demonstrated that this hydrogel could gradually provide oxygen to cardiac cells under ischemic conditions. The results of this study can be used for injuries in which wounds encounter ischemic phenomenon such as diabetic patients [49].

The case of cell-based therapies like stem cells due to their ability to spatter pro-regenerative cytokines, lack of oxygen can fail the treatment by failing transition from the inflammatory step to the proliferation step. Therefore, embedding oxygen along with cells within a polymeric and gel-like layer as a wound dress (for inner and outer injuries) can help cells to access a high concentration of oxygen [47, 50].

Apart from all the above, the oxygen releasing rate is a crucial factor to succeed in advanced wound healing. For instance, when oxygen is delivered too fast, the wound site is overload by oxygen, which leads to **Bohr-effect** (high concentration of oxygen for cells), and part of the oxygen is wasted. On the other hand, if oxygen releasing occurs too slowly, low oxygen concentration results in cell death, wrong signals, and weak cellular functions. So, it is essential to keep oxygen concentration adjusted according to the extracellular matrix and the environment. Programmable hydrogels and biomaterial are known as excellent representatives in this regard [40].

Also, pH is a significant characteristic of the wound healing process. Controlling the oxygen concentration helps to regulate the pH of the wound, especially those in a large size. Less oxygen in the wound site results in forming water and hydrogen peroxide that lowers pH. Scientists could find out that 7.2 < pH < 8.3 is the optimal pH for keratinocytes and fibroblasts cells proliferation in wound healing, which means these cells need a maintained level of pH [51]. Since MBs were introduced as an appropriate means for oxygen transfer, they can also be useful in regulating pH.

To sum up, MBs can strengthen the wound healing chain. Notably, combining MBs with hydrogels enhanced the chance to overcome oxygen diffusion restrictions and experience a fast and advanced wound healing.

## 3 Microbubbles in TE-based treatments

Recent advances in drug delivery systems (DDSs) to release GFs have provided more therapeutic opportunities. Thanks to TE, utilizing biomaterials for medical purposes has introduced new and novel strategies for regenerative medicine. Oxygen, nutrient, and drug supply to implanted tissue scaffolds (In Vitro – In Vivo) has been one of the responsibilities of TE. MBs due to the gaseous core is a robust device not only for oxygen transferring but also for therapeutic agent delivery. Drugs and GFs can be entrapped by MBs in various ways. Merging TE, with MBs in the form of hydrogels and scaffolds, provides significant opportunities in medicine regarding drug and growth factor delivery. Besides, stabilizing charged drugs and GFs onto or on the surfaces of MBs is another role of MBs in DDS. Creating a layer of oil is another way to entrap drugs and GFs in MBs. In this way, the outer surface is supposed to be stabilized and potent against being ruptured. Cationic lipid-coated MBs are positively charged, so they are capable of binding DNA as a polyanion (negatively charged) GFs. During treatments, by employing ultrasound, MBs are exploded, and drugs are released on site.

Drug delivery by biomaterials combined with MBs is hypothesized to be faster than other modern treatments such as lipid nanoparticles, nanofibrous structures, CNTs, and polymeric micro/Nanospheres. Engineered MBs-based biomaterials were developed. Whereby In Vitro experiments, growth factor release and drug delivery to the target site were controlled and enhanced.

On the one hand, decreasing the size of the particles enhances the penetration of the drug into the organ or damaged tissue. On the other hand, by employing **micro modulators** such as MBs, macromolecules can be encapsulated to be protected against environmental factors and have a targeted release on the wound site, tissues, and tumors [52].

In 2008, researchers showed that integrating MBs with TE can promote DDSs and oxygen transfer to the tissue [53]. In this study, a new therapeutic device for oxygen supply to the hypoxic tissues was developed to accelerate the treatment. It was also approved that MBs leave no toxic effects. Passive targeting as a method of drug delivery is known as the most appropriate method for delivering MBs at a targeted site. Drug delivery, regenerating tissue, or killing abnormal cells such as cancer cells have been the focus of scientists and researchers with the aid of MBs. The birth of chemotherapeutic drugs (as potent drugs) by MBs has been the interest of researchers. It is carried out by employing ultrasound after accumulation at the site of the tumor or surgery [54]. Regarding this, scientists could engineer a hydrogel capable of producing MBs to treat bladder cancer using Intravesical Instillation, which has achieved greater efficacy than usual (drug-containing fluid) [42]. Also, in a study by Liao et al. [55], it was shown that a combination of ultrasonic and MBs in cutaneous drug delivery (liquid epidermal growth factor) significantly reduces the time of healing and does not matter if the carrier of MBs is liquid or hydrogel.

MBs could introduce themselves as potent Supplements for **sonodynamic therapy** and antimetabolite therapy in pancreatic cancer therapy [56]. Researchers could employ oxygen-filled MBs to boost targeted treatment of pancreatic cancer. MBs combined with ultrasound and external magnetic, decreased 48.3% of volumes of orthotopic pancreatic tumor after a nine-day treatment compared with the same procedure without MBs [57]. The hydrogel can play a synergistic role by keeping MBs in its structure, whereby MBs accumulation will be under control, which results in advanced treatment [58].

**Figure 5:**
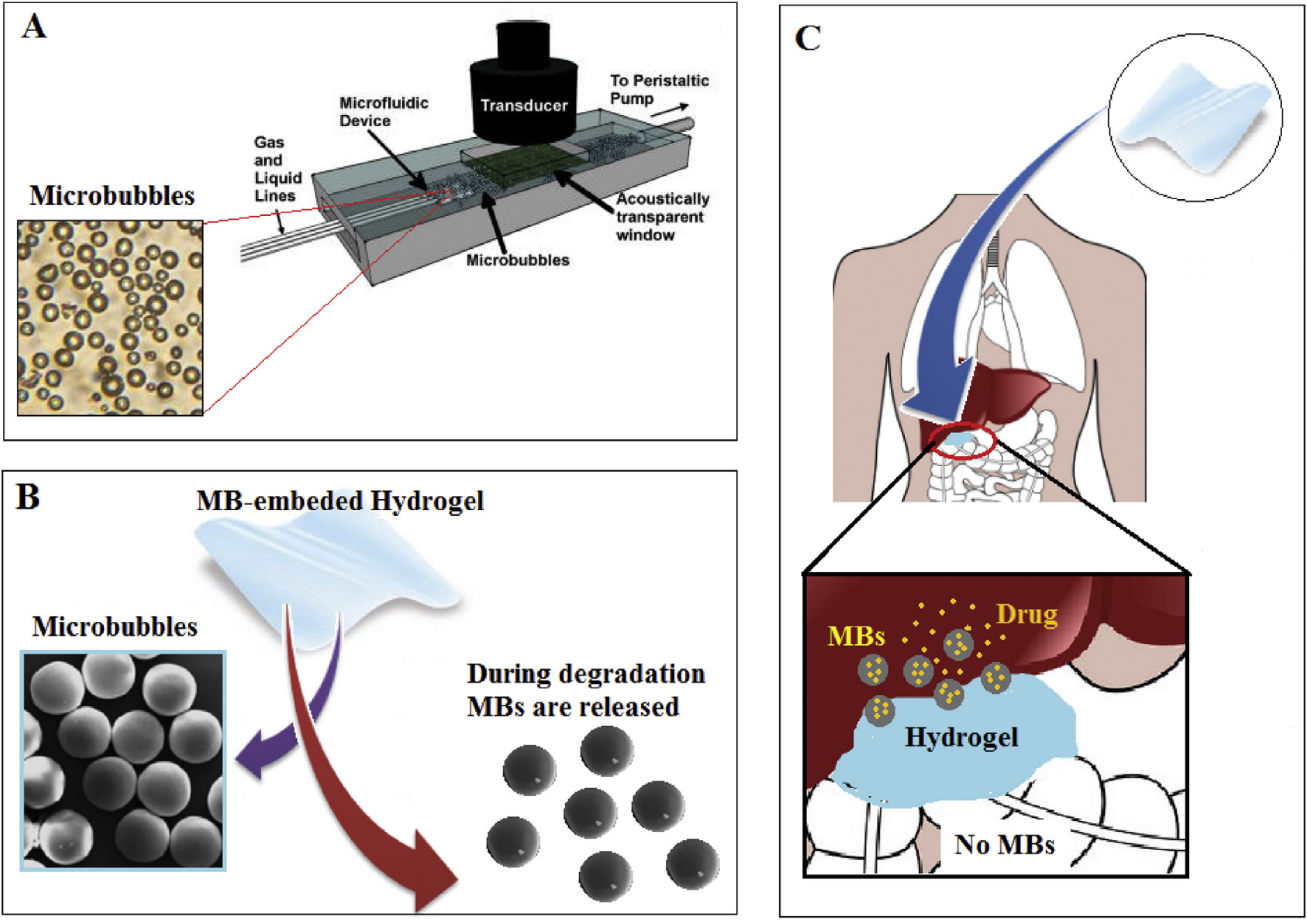
A) Microfluidic system to produce in situ MBs. B) Hydrogels can provide MBs as microfluidic devices. C) Centralized MB releasing by hydrogel prevent side effects.

MBs hold promise as porogen and growth factor carriers, too. Injecting MBs through the blood vessel can elevate the efficacy of the injection-based therapies. Microfluidic fabrication is known as a modern device to advance drug delivery systems and produce inseminated MBs with various kinds of drugs, including chemotherapeutic agents [59]. To overcome atherosclerosis, localized, and targeted drug delivery to the vessel wall simplified with the help of in situ MB production and employing intravascular ultrasound in which intravascular co-injection of MBs and therapeutics was allowed, Figure 5A [60]. Figure 5B depicts that MBs-based biomaterial can be recommended to be used as an in situ MB producer instead of MFDs. Poly (n-butyl cyanoacrylate) MB described their usefulness in both molecular ultrasound imaging and ultrasound-mediated indirect drug delivery by enhancement of drug penetration [61].

In another form of MB usage, hydrogel cellar pregnant with MBs and drugs are promising approaches in DDS. In this way, biodegradable and implantable hydrogel loaded with MBs are embedded in the right spot inside the body or close to the targeted tissue. Once the hydrogel is decomposed (gradually oy suddenly), drug-loaded MBs are released. The positive aspect of this technology is centralized drug delivery, which prevents side effects by avoiding drug-releasing to other organs and tissues, like toxic drugs (e.g., Doxorubicin), which can affect adjacent organs, negatively, Figure 5C. Scientists have employed similar designing of MBs-embedded gel to help the delivery of transdermal Mg ascorbyl phosphate in mice after ultrasound treatment. They highlighted that the viscosity of the medium around affected the survival of MBs with ultrasound treatment. Also, they stated that MBs surrounded by agarose gel apply more easily on the skin surface of live animals [62].

As mentioned previously, oxygen-filled MBs are captured inside a polymeric scaffold loaded with drugs. In this case, MBs play a significant role in oxygen supplying and pH regulation during the healing process. It has been reported that hypoxia condition affects biomaterial degradation rate [63]. Besides, oxygen is fundamental for **epithelization,** metabolic activity, and collagen synthesis [64]. Furthermore, the pH of the extracellular matrix can be altered due to the changes in oxygen level; hence, pH-responsive biomaterials can be programmed by changing MB concentration [65].

MBs can burst and release their content in the tumor sites and kill the tumor cells. One of the most common strategies is to deliver siRNA into tumor cells [66]. Engineering hydrogels containing MBs pregnant with siRNA is a strong DDS to fight against tumor cells [67].

In conclusion, MBs depicted they can be robust but straightforward devices to boost DDS. Their ability to holding and carrying oxygen, drug, and genes give them a strong potential in medical purposes.

## 4 MB in scaffolding

Regarding cell-seeded biomaterials, cells need to increase within the scaffold and move towards the inner zones. On the other hand, cells in the inner sections need nutrients and oxygen, as well as the outer layers. Besides, Scaffolds need to be high permeable towards oxygen. Low diffusion of oxygen through the scaffold is a limiting factor in TE.

Integrating MBs with scaffold fabrication has attracted the scientist's attention. The control of the amount of pore space and uniform porosity, which help cell functions and formation of new tissues and organs, is still an essential challenge in TE. Various methods of fabrication, like electrospinning [68], have their ability to create well porous scaffolds. Embedding porogen within the structure and then removing them by dissolution is another way of creating porosity [69, 70]. MBs can be utilized like porogens but needless to washing step.

**Figure 6:**
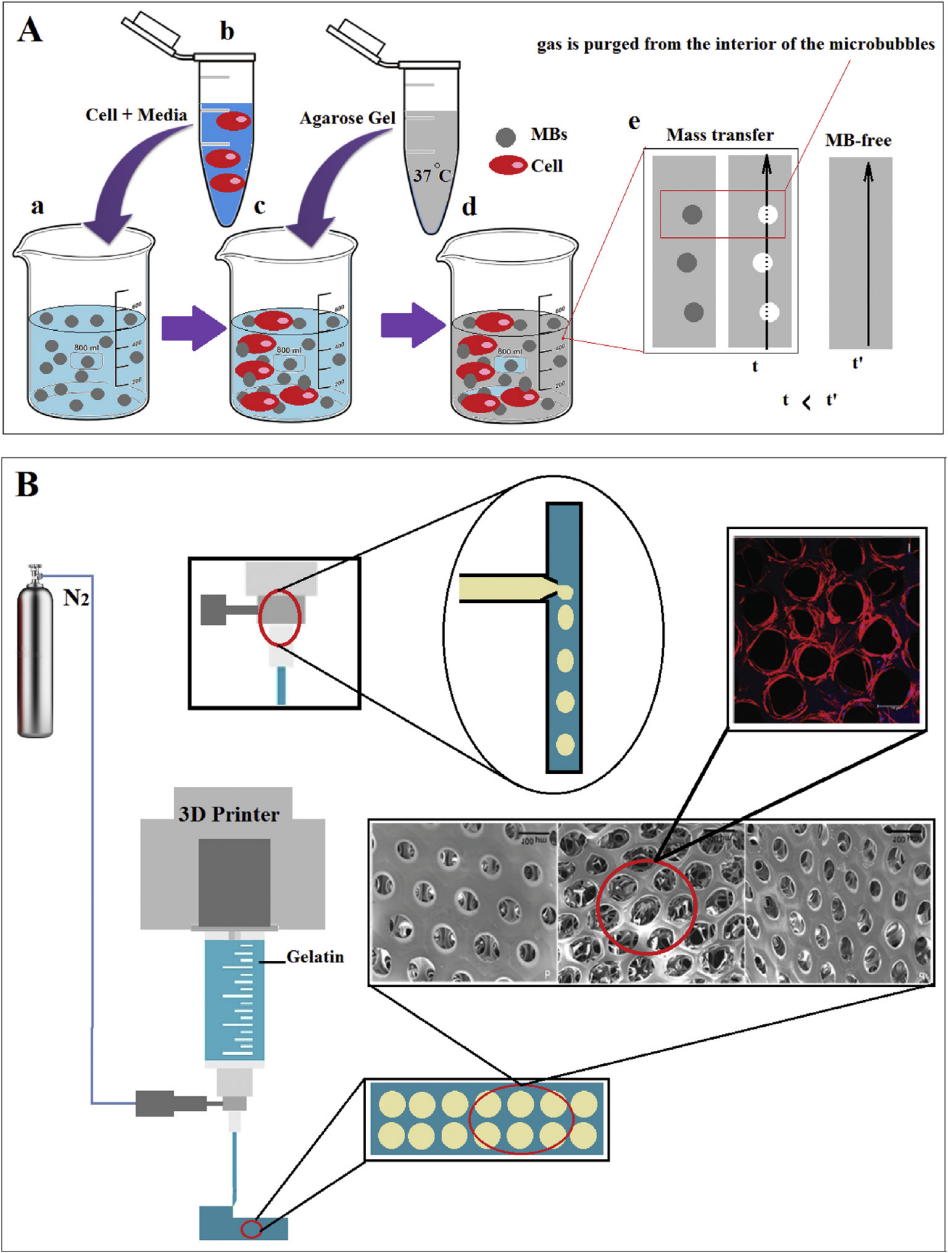
A: a) combination of blend of phosphate-buffered saline and MBs, b) suspension of cell and media, c) microbubble/cell slurry, d) adding Molten agarose to make the final cell-seeded MB scaffold, e) purging the air from the microbubble left pores which accelerates mass transfer through the scaffold. (t: diffusion time for MB-based scaffold, t’: diffusion time for MB-free scaffold). B: Employing MB in 3D printing fabrication to create porous scaffolds.

Scaffolds embedded with MBs demonstrated a higher mass transfer through its structure in contrast with the MB-free buildings. Lima et al. [71] engineered a building having high potential in mass transfer. They first prepared a blend of phosphate-buffered saline and MBs (Figure 6-Aa) and then combined it with the cell media (Figure 6-Ab) to have microbubble/cell slurry (Figure 6-Ac). Finally, molten agarose was added to make the desired cell-seeded microbubble scaffold (Figure 6-Ad). They predicted that the time of diffusion would be no longer than the typical MB-free scaffold (Figure 6-Ae). MBs enhance the media content of the building, which helps cells to feel happy to grow. Non-vascularized scaffolds can be improved by MBs recruitment. It has been inferred that better angiogenesis and a well-vascularized tissue will be achieved if MBs be Scattered within the scaffold [72].

The mechanical properties of the final printed scaffold are essential, as well as porosity. The strength, tension, and surface roughness of the scaffold can be altered. The type of gas (e.g., inert gas) has a significant role in the desired mechanical characteristics. It was approved that tensile strength and surface roughness of the final gas-assisted 3D printed scaffold was improved by employing MBs [73].

Scaffolds with homogenous and high porosity can be fabricated through the microfabrication process, too. In previous studies, the generation of bubbles and various polymeric porous membranes by using a T-junction MFDs have been carried out and utilized for tissue scaffold fabrication [74, 75]. Bayram et al. [76] worked on a new way to fabricate 3D gelatin tissue scaffolds with uniform and interconnected pores. MBs generated via T-Junction microfluidics played a vital role in making uniform and high porosity.

MBs have further applications in case of scaffolding. They are used for cell culture applications and enhance the transport and uptake of drugs. Regarding this, they have a highly hydrophobic gas core (e.g., SF6, C3F8, or C4F10) stabled by a shell of phospholipids, albumin, or polymers. As mentioned in the previous section, MBs can carry drug molecules, DNA fragments, or nanoparticles [77].

Supplying a sufficient amount of oxygen deep into the densely cultured cells within the scaffold is another challenging issue in TE. MBs proved their capability as oxygen-carrier, too. This study revealed that MBs were able to increase the **osteoblastic cell** activity in scaffolds (In Vitro experiments) [78].

MBs-scaffolds and mouse pulmonary stem/progenitor cells (mPSCs) were used to produce alveoli-like structures that the regenerated cartilage successfully [81]. It was approved that MBs-scaffold will provide a microenvironment for mPSCs to differentiate into pneumocystis and to recruit blood vessel formation. Embedding MBs within the scaffold could also improve the process of lung TE because of using angiogenesis and pneumocystis differentiation in alveogenesis [79]. MBs showed that they could boost and accelerate cell differentiation, but discrepancy amongst the size of MBs can alter cell differentiation rate.

A gelatin MBs-based Poly (DL-lactic acid-co-glycolic acid) scaffold has been engineered for bone TE application. Embedding MBs in the scaffold demonstrated better cell attachment and proliferation and controlled the release rate of cytokines. In this research, MBs revealed a core-shell structure with a protein shell around the nitrogen gas core [80].

### 4.1 MB in 3D printing

3D printing (3DP) as a novel fabrication method (nicely reviewed by Jingyu Liu and Cheng Yan [81]) is employed along with MBs in two ways. i) 3DP used for MBs generation by making MFDs, and ii) MB-assisted 3D printing. The aim is to engineer scaffolds with high potential in cell differentiation, adhesion, angiogenesis, degradation, and mechanical characteristics.

MBs can be mixed directly with cells and uncross-linked biomaterials before 3DP. Depending on the process, MBs can remain for a while or collapse or merge or dissolve [71]. Scientists could design MFDs that they were able to produce MBs in micrometer-sized channels (range of 1.0–10.0 μm). 3DP, due to its operational simplicity, has been a viable alternative to engineer MFDs. Walter Araujo Filho could manufacture a MFDs by using 3DP to produce MBs having the olive oil as the coating layer, for drug-carrying purposes [82].

The porous biomaterials can affect cell behavior within the artificial environment. In a study by Kuan-Han Wu et al. [83], dealing with the self-repairing of cartilage tissue, a gelatin MBs-scaffold was manufactured using 3DP. They showed that pore size influences cell growth and differentiation (Figure 6B). They proved that MBs with a size of 200 μm in diameter results in the best chondrogenesis results. Similarly, Lipid-coated MBs demonstrated that it is capable of enhancing the osteogenic differentiation of mesenchymal stem cells in 3D printed scaffolds under low-intensity pulsed ultrasound [84]. Furthermore, highly ultrasound responsive Gas-filled MBs encapsulated with lipid revealed robust potential in increasing the number of human mesenchymal stem cells in a 3D printed poly (ethylene glycol) diacrylate scaffold [85].

The reason for this stimulation is attributed to the useful role of MBs as ultrasound contrast agents. Programmed scaffolds can be engineered by employing MBs. In the case of degradation, polymers and hydrogels can be processed by changing their porosity by embedding MBs within their structure. 3D printers equipped with an MB generator create high porous scaffolds.

In general, Gas-assisted scaffolding techniques improved their mechanical and biological characteristics of the conventional scaffolds by altering the porosity of the scaffolds.

## 5 Concerns and safety

MBs revealed their ability in various aspects of TE and depicted satisfactory results during treatments and processes. For instance, they proved that they could be employed as a potent vehicle in medicine regarding their ability to carry drugs. One of the positive features of MBs relay on their nonchemical essence. They can be combined with biomaterials with leaving no cytotoxicity. MBs produce simple, safe, and strong emissions when embedded in biological materials, including hydrogels. Nanoporosity created in Scaffolds by MBs caused a robust mass transfer with no side effects. Scaffolds created with MBs as porogens demonstrated biocompatible behavior [71]. In the case of cancer drug delivery, side effects of chemotherapy drugs injected in the bloodstream circulation system go under control by entrapping them in MBs.

Also, it must be mentioned that MBs decrease the dosage of drugs during treatments [1]. MBs can be loaded with Different types of ligands like antibodies as receptors to make a targeted delivery.

Despite the potential medical applications of MBs, they can cause side effects. In the literature, there were reports about hurting tissue or vessels and capillaries as a result of the usage of MBs. However, one of their benefits is avoiding damaging to untargeted organs and tissues. In treatments such as cardiac surgery, the use of MBs, flow can damage the vessels and capillaries of the surgical site. Besides, the longevity of the MBs, especially within the circulation system, can be considered as a limitation item. MBs must reach the desired site inside the body and then burst; otherwise, they may hurt other parts of the body, thereby the shell of MBs can be considered as a limiting factor during drug delivery [86] and needs to be assessed and engineered according to studies. Generally, regarding In Vitro studies, MBs seem to be safe when they are incorporated in TE. In contrast, in the case of In Vivo studies, side effects must be considered, mainly those that include combining biomaterials with ultrasound.

## 6 Conclusions and future perspectives

MBs are gas bubbles with a diameter of <50 μm, mostly designed to carry gas, drugs, and GFs. MBs have been able to attract the researcher's attention in TE as well as other medical strategies.

MBs could improve different aspects of TE, including Wound healing systems, DDS, and scaffolding. MBs showed that they could be useful if be combined with biomaterials. They also presented: i) an accurate and targeted drug delivery, ii) a fast and clean wound healing and also novel bondages, iii) elevation of the mechanical, and physiobiological properties of the scaffolds and iv) providing better and in-depth mass transfer within the scaffold.

We strongly believe in effort and non-stop perseverance in science. Thanks to all authors of the literature that we cited their study in our paper, MBs are now one of the robust devices that can facilitate and promote therapies in the future by controlling their size and concentration. Eventually, employing MBs in preclinical studies and treatments may be more productive and significant than their diagnostic usage. Furthermore, Using MBs can restrict employing chemical reagents as fillers or porogens, thereby cells will experience less toxic environments. According to our research, few articles were published regarding utilizing MBs in TE, especially in wound healing strategies (combined with hydrogels) and scaffolding. Although MBs have proven their ability in medical and TE approaches, they can be harmful if not used properly. There are still challenges and therapeutic problems that need further investigation regarding MBs, and there is a hope that researchers try to employ MBs in their future studies.

We provided a short review of MBs application in TE that we hope opens new doors to tissue/biomedical engineers in order to employ them in their interests to overcome weaknesses of the current therapies.

## Declarations

### Author contribution statement

All authors listed have significantly contributed to the development and the writing of this article.

### Funding statement

This research did not receive any specific grant from funding agencies in the public, commercial, or not-for-profit sectors.

### Competing interest statement

The authors declare no conflict of interest.

### Additional information

No additional information is available for this paper.
